# Adaptation of High-Growth Influenza H5N1 Vaccine Virus in Vero Cells: Implications for Pandemic Preparedness

**DOI:** 10.1371/journal.pone.0024057

**Published:** 2011-10-13

**Authors:** Yu-Fen Tseng, Alan Yung-Chih Hu, Mei-Liang Huang, Wei-Zhou Yeh, Tsai-Chuan Weng, Yu-Shuan Chen, Pele Chong, Min-Shi Lee

**Affiliations:** National Institute of Infectious Diseases and Vaccinology, National Health Research Institutes, Miaoli, Taiwan; Johns Hopkins University - Bloomberg School of Public Health, United States of America

## Abstract

Current egg-based influenza vaccine production technology can't promptly meet the global demand during an influenza pandemic as shown in the 2009 H1N1 pandemic. Moreover, its manufacturing capacity would be vulnerable during pandemics caused by highly pathogenic avian influenza viruses. Therefore, vaccine production using mammalian cell technology is becoming attractive. Current influenza H5N1 vaccine strain (NIBRG-14), a reassortant virus between A/Vietnam/1194/2004 (H5N1) virus and egg-adapted high-growth A/PR/8/1934 virus, could grow efficiently in eggs and MDCK cells but not Vero cells which is the most popular cell line for manufacturing human vaccines. After serial passages and plaque purifications of the NIBRG-14 vaccine virus in Vero cells, one high-growth virus strain (Vero-15) was generated and can grow over 10^8^ TCID_50_/ml. In conclusion, one high-growth H5N1 vaccine virus was generated in Vero cells, which can be used to manufacture influenza H5N1 vaccines and prepare reassortant vaccine viruses for other influenza A subtypes.

## Introduction

Outbreaks of avian influenza H5N1 viruses emerged in 1997 and are still killing avian hosts and causing zoonotic transmission to humans in 2011, posing the persistent threat of influenza pandemics in humans [1]. Vaccination is the most cost-effective strategy to control and prevent influenza pandemics and seasonal epidemics. Most current seasonal influenza vaccines are manufactured using chicken embryonated eggs, which is labor-intensive and hard to scale up during a pandemic. Moreover, egg supply may not be available during a pandemic causing by H5N1 viruses that are highly pathogenic to chickens. Therefore, the World Health Organization (WHO) has been encouraging the development of cell-based influenza H5N1 vaccines since 2006 [2].

Two cell lines, Vero and MDCK cells, have been licensed for manufacturing influenza vaccines [3], [4], [5], [6]. In addition to influenza vaccines, Vero cells have been widely approved for manufacturing other human vaccines but MDCK cells are only licensed for influenza vaccines. Currently, there are four clades of influenza H5N1 viruses circulating in avian hosts and causing zoonotic transmission to humans. Therefore, the WHO have collaborated with reference labs to prepare vaccine seed viruses from representative viruses of the two clades for vaccine development and production [1]. The current clade-1 influenza H5N1 vaccine strain (NIBRG-14), provided from the UK NIBSC (National Institute for Biological Standards and Control), is a reassortant virus containing NA and modified HA gene segments of A/Vietnam/1194/2004 (H5N1) virus and the other 6 gene segments of egg-adapted high-growth A/PR/8/1934 (H1N1) virus [7]. The NIBRG-14 vaccine virus could grow to high titers in chicken eggs and MDCK cells but not Vero cells which is the most popular cell line for manufacturing human vaccines [8], [9], [10], [11]. Historical studies have shown that high-growth influenza viruses can be selected through continuous passages and plaque purifications in eggs and cells [12], [13]. This study was conducted to adapt the NIBRG-14 vaccine virus to grow efficiently in Vero cells.

## Results

### Growth curve of NIBRG-14 and Vero-adapted viruses

**Figure 1:**
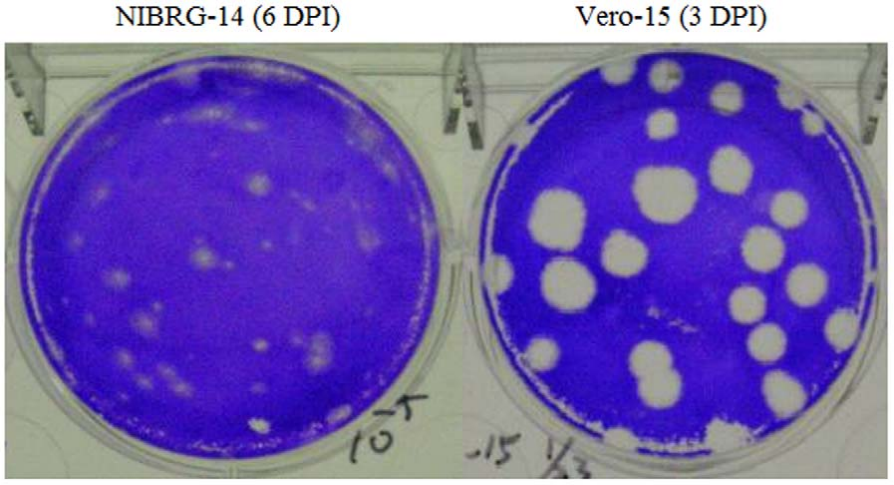
Plaque morphology of NIBRG-14 and Vero cell-adapted (Vero-15) H5N1 viruses grown in Vero cells in different days post infection (DPI).

The NIBRG-14 viruses could only form small and ambiguous plaques after 6 days post infection in Vero cells (Figure 1) and reach peak infectious virus titers about 10^5.5^ TCID_50_/ml (Table 1). After three passages, several big plaques were observed so a plaque purification was conducted at the fourth passage. Another plaque purification was conducted at the sixth passage. Overall, after 11 passages including 2 times of plaque purifications in Vero cells, one high-growth virus clone (Vero-15) was selected. The Vero-15 virus can form clear and big plaques after 3 days post infection in Vero cells (Figure 1) and reach peak infectious virus titers about 10^8^ TCID_50_/ml (Table 1). The Vero-15 virus was further used to produce virus stocks in T-flasks with high virus titers (1.9×10^8^ TCID_50_/ml and 1.4×10^8^ PFU/ml) for further evaluations including genetic and antigenic characterization and pilot production in microcarrier-based cell cultures.

**Table 1:** Growth efficiency of the NIBRG-14 and Vero-adapted influenza H5N1 (Vero-15) viruses with different multiplicity of infection (MOI) in T-flasks.

	NIBRG-14 (MOI = 10^−2^)	NIBRG-14 (MOI = 10^−3^)	Vero-15 (MOI = 10^−3^)	Vero-15 (MOI = 10^−4^)
Days post-infection	TCID_50_/ml (log_10_)	TCID_50_/ml (log_10_)	TCID_50_/ml (log_10_)	TCID_50_/ml (log_10_)
1	<2	<2	4.33±0.07	3.71±0.10
2	<2	<2	6.80±0.17	7.80±0.24
3	4.16±0.13	4.93±0.29	**7.03±0.23**	**8.30±0.13**
4	4.42±0.07	4.78±0.52	not available	not available
5	**4.55±0.13**	**5.53±0.03**	not available	not available

TCID_50_ (50% tissue culture infectious dose) was measured in triplicate and shown as geometric mean ± standard error of mean.

Boldfaces indicate peak infectious virus titers.

### Antigenic and genetic characterization

Using the NIBRG-14 standard sheep antisera, HI antibody titers against the NIBRG-14 and Vero-15 viruses were 800 and 400, respectively. The results indicate these two viruses have similar antigenicity. Compared with the NIBRG-14 strain, the Vero-15 strain does not have any nucleotide difference in HA, NA, NP and M gene segments but has 1–4 nucleotide differences in PB2, PB1, PA and NS gene segments (Table 2). The only amino acid change in PB2 protein between the NIBRG-14 and Vero-15 viruses was S→Y at position 360 (based on PR8 numbering), which is not likely related to the well-defined marker of pathogenicity at position 627 [14].

**Table 2:** Genetic differences between the NIBRG-14 and Vero-adapted H5N1 (Vero-15) viruses.

	Nucleotides changes			Amino acid changes		
Gene segments	Position	NIBRG-14	Vero-15	Position	NIBRG-14	Vero-15
PB2	1077	c	a	360*	S	Y
PB1	583	a	t	195	K	L
	584	a	t			
	1257	c	t			
	1737	g	a			
PA	1482	a	g	494	E	G
NS	271	t	c	90	L	P
	331	t	c	110	L	P
	335	t	c			

*Based on numbering of A/PR/8/34 (accession no. CAA23855).

### Virus growth in microcarriers

Using 5 g/L of microcarriers and serum-containing medium in 100-ml scale spinner flasks, cells grew to confluence (∼2×10^6^ cells/ml) at day 4 or 5. After infected with the Vero-15 viruses under MOI of 0.0001, infectious virus titers sharply increased at day 3 and peaked at day 5 (10^8.78^ TCID_50_/ml). The HA titers had the similar pattern (Table 3).

**Table 3:** Growth efficiency of Vero-adapted influenza H5N1 virus (Vero-15) in microcarrier-based Vero cell cultures in 100-ml spinner flasks.

	Virus titers	
Days post infection	HA units/50 µL	TCID_50_/ml (log_10_)
2	<4	3.83±0.19
3	128	7.71±0.10
4	1024	8.58±0.07
5	1024	8.78±0.10
6	2048	8.60±0.00

HA: hemagglutination.

TCID_50_ (50% tissue culture infectious dose) was measured in triplicate and shown as geometric mean ± standard error of mean.

## Discussion

It is well known that wild-type influenza viruses and egg-adapted high-growth reasortant influenza vaccine viruses can grow efficiently in MDCK cells but not Vero cells [3], [15]–[18]. After 20 passages of an egg-adaptedd high-growth reassortant H1N1 vaccine virus in Vero cells, the titer of infectious virus increased 3 (7.18 to 7.70 log_10_ PFU/ml) to 26 (6.95 to 8.37 log_10_ TCID_50_/ml) folds and their viral antigenicity and HA sequences were stable in a previous study [15]. In our study, we adapted an egg-based high-growth reassortant H5N1 vaccine virus after 11 passages in Vero cells including 2 times of plaque purification. Our Vero cell-adapted H5N1 virus increased over 100 folds in infectious virus titers measured by TCID_50_ and plaque assay without changing antigenicity and HA sequences.

Based on statistics from the WHO in March 2008, more than 70 clinical trials of pandemic influenza vaccines have been completed or are ongoing (www.who.int). Since most vaccine manufacturers used the egg-based technology for production of seasonal influenza vaccines and converted the egg-based facility to manufacture influenza H5N1 vaccines for clinical trials, only 6 clinical trials used cell-based vaccines. Most of the 6 trials using cell-derived vaccines were based on MDCK cells and one trial was based on Vero cells. As the NIBRG-14 virus can not grow efficiently in Vero cells, wild-type A/Vietnam/1203/2004 (H5N1) was used to manufacture vaccines in Vero cells in facility under biosafety level 3 [10], which is not feasible in most developing countries. In our study, a high-growth H5N1 virus was generated in Vero cells. Ideally, antigenicity of influenza vaccine strains should be tested using ferret antisera, which are not readily available. Alternatively, we used the NIBSC sheep standard antisera to perform the antigenicity test. Moreover, the Vero-adapted H5N1 virus has the same HA sequence with the NIBRG-14 virus, which further indicated the same antigenicity between the Vero-adated H5N1 and NIBRG-14 viruses. We further demonstrated commercial potential of the Vero-adapted H5N1 virus in the microcarrier-based cell culture system reaching peak virus at 10^10^ TCID_50_/ml which is higher than that of the NIBRG-14 virus in microcarrier-based MDCK cell cultures (10^8–9^ TCID_50_/ml) [9]. Scale-up production and process development in the microcarrier-based Vero cell culture system are ongoing.

In addition to being used as the seed virus for manufacturing H5N1 vaccines, the Vero-adapted virus has the potential to become a master virus to generate high-growth reassortants for other influenz A viruses. The current egg-based technology for vaccine production is heavily relied on the high-growth PR8 master virus for generating seed viruses [12]. Therefore, it would be desirable to establish high-growth master viruses for Vero cell-based vaccine production [14], [15], [16], [17], [18], [19]. Several studies have proved the feasibility of generating reassortant vaccine viruses in Vero cells [7], [19], [20], [21]. We are using the Vero-15 virus as a master virus to generate reassortant viruses for other influenza A subtypes and evaluate their growth efficiency in Vero cells.

It has been reported that antigen yields of engineered-H5N1 viruses in eggs are 30–40% lower than the average of seasonal influenza vaccines [22]. Howard et al., found that recent wild-type H5N1 viruses (A/Vietnam/1203/94 and A/Indonesia/05/2005) grew to 0.2 to 1×10^9^ TCID_50_/ml in microcarrier-based Vero cell culture systems [10], which is similar to the virus titers of our Vero cell-adpated H5N1 reassortant virus but is much higher than those of seasonal wild-type influenza viruses and egg-adapted vaccine viruses [3]. In addition, these wild-type H5N1 viruses did not change their antigenicity and HA sequences in the end of production [10]. Moreover, this H5N1 vaccine candidate was well-tolerated and highly immunogenic in humans and has been licensed as a prepandemic vaccine in Europe [11], [23]. The reasons why the wild-type H5N1 viruses grow much efficiently than wild-type H1N1 and H3N2 viruses are not clear, which need further studies to elucidate.

The egg-adapted high-growth A/PR/8/1934 (PR8) virus strain has been widely used as a master donor virus to generate vaccine seed viruses for manufacturing egg-based inactivated influenza vaccines [7], [12]. One recent study showed that polymerase (PA/PB1/PB2) and NS gene segments were related to high-growth property in eggs of the PR8 virus [8]. In addition, high-growth property in MDCK cells of PR8 virus was determined to be related to single mutation at position 360 (S→Y of PB2 protein [24]. A previous study suggested that NS gene is related to adaptation of the PR8 virus in Vero cells but molecular determinants of high-growth influenza viruses in Vero cells have not been well defined [19]. Our Vero-adapted influenza H5N1 virus has mutations on 4 gene segments including the S360Y mutation of PB2 protein, compared with the original NIBRG-14 H5N1 vaccine virus. Identifying molecular determinants of high-growth influenza viruses in Vero cells would be valuable for establishing the platform of Vero cell-based influenza vaccine production.

The current global manufacturing capacity of influenza vaccines are concentrated in Australia, Europe and North America and will not meet the urgent demand during an influenza pandemic, so countries without manufacturing capacity may not be able to have pandemic influenza vaccine available [2], [25], [26]. Therefore, WHO has developed a global pandemic influenza action plan to increase vaccine supply. Building new production plants in both developing and industrialized countries is one of the various strategies suggested in the WHO action plan [2]. Egg-based and cell-based culture systems are two matured technologies for manufacturing influenza and other viral vaccines. The egg-based technology has long history of success on supplying seasonal influenza vaccines but it can not be scaled-up in a short term and may not have egg supply during a pandemic causing by highly pathogenic avian influenza viruses, such as H5N1 viruses. Therefore, cell-based technology is becoming an attractive option for new facilities. A novel influenza H1N1 virus emerged in March 2009 in Central America and spread globally by June 2009 [26], [27]. Among the various tools to mitigate the impact of a pandemic, vaccination is considered to be the most cost-effective strategy. The urgent development of pandemic influenza H1N1 vaccines raised complex challenges, especially at a time when seasonal vaccine productions were ongoing. Therefore, pandemic influenza H1N1 vaccines were not available until September 2009 and their supplies were far below the expected productivity [26], [27]. Since Vero cell is the most popular continuous cell line for manufacturing human vaccines and Vero cell banks fulfilling the cGMP requirements are readily available in many vaccine production facilities, WHO and national authorities may consider preparing for the next influenza pandemic by generating Vero cell-adapted high-growth vaccine viruses with pandemic potentials such as influenza H2N2, H5N1, H7N7 and H9N2 and conducting clinicl trials of these Vero cell-derived panemic vaccine candidates. When the next influenza pandemic occurs, all Vero cell culture facilities can be conveted to manufacture pandemic influenza vaccines. Thus, global productivity of pandemic influenza vaccines will be increased promptly to meet the urgent demand during influenza pandemics.

## Materials and Methods

### Adaptation of NIBRG-14 in Vero cells

The NIBRG-14 vaccine viruses were grown in eggs by the NIBSC and supplied to Taiwan Centers for Disease Control (CDC). Taiwan CDC further amplified the NIBRG-14 viruses in eggs and supplied this to the Vaccine Research and Development Center of National Health Research Institutes (NHRI), Taiwan. Vero cells were purchased from the American Type Culture Collection (ATCC, USA) and were grown in M199 medium (GibcoBRL, USA) plus 10% FBS (Moregate, Australia) within passage 135–150. The NIBRG-14 vaccine viruses were adapted with 11 passages in Vero cells supplemented with 2 µg/ml TPCK-trypsin (Sigma, USA) including 2 rounds of plaque purification in 6-well plates. The infectious virus research was conducted under biosafety level 2+ approved by the NHRI Biosafety Office.

### Virological assays

HA titrations were conducted in 96-well microplates using turkey red blood cells (RBC) following the standard technique [28]. Virus infectious titers were measured using the 50% tissue culture infectious doses (TCID_50_) assay based on cytopathic effect (CPE) and the plaque assay based on plaque forming unit (PFU) in Vero cells [15], [28]. A positive control with pre-specified acceptable range was included for conducting HA, TCID_50_, and plaque assays.

### Determination of virus growth efficiency in Vero cells

Influenza H5N1 viruses were grown in T75 flasks supplemented with TPCK-trypsin and supernatant was harvested at day 1–5 post infection for measuring HA and infectious virus titers. For the NIBRG-14 virus strain, multiplicity of infection (MOI) were 10^−1^, 10^−2^, and 10^−3^ TCID_50_/cells since the NIBRG-14 virus strain can not cause significan CPE in a lower MOI. For the Vero-adapted virus strain, MOI were 10^−3^, and 10^−4^ TCID_50_/cells.

### Antigenicity test

To measure the antigenicity relatedness between NIBRG-14 and the Vero cell-adapted viruses, NIBRG-14 standard antisera (NIBSC, UK) was used to measure antibody titers against NIBRG-14 and Vero cell-adapted viruses using the standard hemagglutinination inhibition (HI) assay [28].

### Determination of virus nucleotide sequences

Virus RNA was extracted by a commercial kit (Geneaid, Taoyuan, Taiwan). The extracted virus RNA was amplified using the one-step RT-PCR (Promega, Madison,WI) for HA and NA or two-step RT-PCR (Invitrogen, USA) for the other six gene segments. Sequences of the oligonucleotide primers used in this study are available upon request. The amplified DNA was sequenced using the ABI 3730 XL DNA Analyzer (Applied Biosystem Inc., Foster City, CA). Nucelotide sequences reported in this study have been submitted to public domain (accession no. GQ454861∼GQ454868).

### Cell and virus culture in microcarriers

Potential of using the Veo-adapted H5N1 viruses as a vaccine seed virus was evaluated in a microcarrier-based cell culture system using serum-containing medium. Cytodex 1 microcarriers (GE Healthcare, USA) were hydrated, autoclaved, and preconditioned according to manufacturer's instruction. Growth curves of Vero cells with different culture conditions were tested in 100-ml spinner flasks (50 ml working volume) and microcarriers were sampled to count cell density every day. When the cells grew confluent on microcarriers, the H5N1 viruses were added to infect cells cultured in M199 medium supplemented with TPCK-trypsin [9].
